# Do You Know What I Know? The Impact of Participant Role in Children's Referential Communication

**DOI:** 10.3389/fpsyg.2016.00213

**Published:** 2016-02-23

**Authors:** Holly P. Branigan, Jenny Bell, Janet F. McLean

**Affiliations:** ^1^Department of Psychology, The University of EdinburghEdinburgh, UK; ^2^Division of Psychology, Abertay UniversityDundee, UK

**Keywords:** children, dialogue, common ground, referential communication, participant role

## Abstract

For successful language use, interlocutors must be able to accurately assess their shared knowledge (“common ground”). Such knowledge can be accumulated through linguistic and non-linguistic context, but the same context can be associated with different patterns of knowledge, depending on the interlocutor's participant role (Wilkes-Gibbs and Clark, 1992). Although there is substantial evidence that children's ability to model partners' knowledge develops gradually, most such evidence focuses on non-linguistic context. We investigated the extent to which 8- to 10-year-old children can assess common ground developed through prior linguistic context, and whether this is sensitive to variations in participant role. Children repeatedly described tangram figures to another child, and then described the same figures to a third child who had been a side-participant, an overhearer, or absent during the initial conversation. Children showed evidence of partner modeling, producing shorter referential expressions with repeated mention to the same partner. Moreover, they demonstrated sensitivity to differences in common ground with the third child based on participant role on some but not all measures (e.g., description length, but not definiteness). Our results suggest that by ten, children make distinctions about common ground accumulated through prior linguistic context but do not yet consistently deploy this knowledge in an adult-like way.

## Introduction

Learning to use language successfully requires more than simply acquiring words to express particular concepts and the grammar to combine those words to form particular propositions; it also involves learning when to use which words and which grammatical forms to particular listeners so that the speaker's meaning is appropriately communicated to the addressee. Adults appear to use information from a range of sources to shape the way in which they design their utterances to be easily understood. Research with children suggests that they begin to show sensitivity to a conversational partner's perspective in their language use from an early age, but it is still unclear what factors they take into account when modeling their partner's knowledge, and exactly how such beliefs about their partner's knowledge are manifested in their language production. In this research, we consider whether 8 to 10-year-old children are able to draw appropriate inferences about their partners' knowledge on the basis of their partners' participation in previous dialogue, and examine how such inferences might be reflected in the language they produce.

Speakers can refer to things in many different ways; for example, the same entity can be described as *a dog* or *the fluffy Labrador from down the road.* This is particularly the case for entities with low codability such as tangrams, which can usually be conceptualized in very different ways (e.g., as a skater vs. a chicken) depending on a speaker's perspective (Clark and Wilkes-Gibbs, 1986). How then does a speaker choose a particular referring expression to use? Substantial research has suggested that speakers' choices involve *audience design* (Bell, 1984), or a consideration of what the addressee is likely to understand. To do this, speakers draw on their *common ground*, the knowledge that they believe themselves to share with their listeners.

Clark and Marshall (1981) identified three possible sources of shared knowledge. One important source is beliefs about the cultural communities to which their listeners belong (Fussell and Krauss, 1992). For example, if the speaker believes that she and the addressee are both members of the University of Edinburgh community, she can assume that they share knowledge about particular buildings, people, procedures, and so on. Adults consistently use such beliefs to choose between alternative referring expressions (e.g., whether to refer to a building as “*McEwan Hall”* vs. “*The round building with the dome”*; Isaacs and Clark, 1987).

But assumptions about shared knowledge can also be based on evidence that is tied to particular interactions. Speakers can make reference to the physical context in which they and their listeners are situated, and assume that an object (or indeed any kind of experience) that is physically co-present, and of which listeners might be aware, constitutes part of their common ground. Similarly, they can make reference to previous physical co-presence (e.g., common past experiences).

More relevantly for our concerns, they can also make reference to preceding linguistic context, in other words the language that the speaker and listener have previously used together (in the current or previous conversations), and the meanings that they have jointly established for these utterances. Thus when a speaker produces an utterance in the presence of a particular listener (e.g., “*This tangram looks like a chicken”*), its linguistic content (e.g., words, syntax, phonology) becomes part of their linguistic common ground. In addition, their shared understanding of the meaning of this utterance (the *situation model* that it maps onto; Zwaan and Radvansky, 1998) becomes part of their linguistic common ground.

However, Clark and Wilkes-Gibbs (1986) suggested that the reference of an utterance (i.e., the link between the linguistic expression and the particular referent to which it is intended to refer) becomes part of common ground only following a collaborative process that requires the participation of both speaker and addressee to establish a mutual belief that the addressee has correctly understood the speaker's intended reference. Only when the speaker and addressee mutually accept that the addressee has understood the speaker sufficiently can the reference enter their common ground. Once this mutual acceptance has been reached, the speaker can subsequently assume that the addressee will understand that reference correctly if she uses it again. The speaker and addressee therefore form a *referential pact* for how to refer to the object (e.g., as a chicken).

Accordingly, Clark and Wilkes-Gibbs (1986) showed that when speakers (*Directors*) described a set of tangrams to the same partners (*Matchers*), they initially tended to produce extended descriptions and indefinite references (e.g., “*looks like a person who's ice skating, except they're sticking two arms out in front”*), which were shaped by feedback from their partners over a number of turns (just 18% of initial descriptions were immediately accepted by the Matcher, in what Clark and Wilkes-Gibbs termed a *basic exchange*) until both participants were satisfied that understanding had been achieved. When they subsequently referred to the same tangrams, speakers tended to use definite and considerably shorter references (e.g., “*the ice skater”*), and addressees were able to accept these without requiring further elaboration. Brennan and Clark (1996) showed that speakers also produced fewer *hedge* expressions (indicating provisionality; e.g., *sort of, a bit*) on repeated reference. The result of these adaptations was that communication became faster and more efficient, requiring fewer words and fewer turns.

These findings suggest that speakers' choice of referring expressions was affected by their previous discourse with a partner (see also Garrod and Anderson, 1987). Brennan and Clark (1996) subsequently showed that these effects were partner-specific: Speakers used the same referring expressions repeatedly with the same partner, even when the context made them over-informative. Referential pacts also affect comprehension, with addressees showing slower reaction times to identify referents when the speaker violates a referential pact by using a new term for a referent, even if it is otherwise an appropriate description (e.g., Metzing and Brennan, 2003; Shintel and Keysar, 2007; Brown-Schmidt, 2009a).

Clark and Carlson (1982) noted that dialogues may also involve roles other than speaker and addressee. For example, a person may be a ratified participant in a conversation, but not be directly addressed by the speaker. Clark (1992) proposed that such *side participants* accrue common ground in the same way as speakers and addressees; they share responsibility for tracking what is said and for ensuring that they understand the speaker. The speaker can therefore assume that anything that forms part of their common ground with an addressee also forms part of their common ground with a side participant. In contrast, *overhearers* are not ratified participants in the conversation: Although they hear what the speaker says, they are not under any responsibility to maintain a record of the discourse or to ensure that they have understood the speaker (and by corollary, do not have privileges to collaborate to reach understanding). They do not therefore accumulate common ground with the speaker in the same way as the addressee, and the speaker cannot assume that overhearers have access to the same common ground as an addressee. In accord with this proposal, Schober and Clark (1989) showed that overhearers had a poorer understanding of a director's descriptions in a tangram task than addressees, even when they heard the entire dialogue and were given the advantage of being able to pause and replay the director's descriptions, suggesting that they did not have access to the same common ground as addressees.

Wilkes-Gibbs and Clark (1992) showed that such differences in common ground associated with different participant roles were reflected in speakers' referential behavior. Speakers repeatedly described a set of tangrams to a partner (as in Clark and Wilkes-Gibbs, 1986; Matcher A), before describing the same set to a different partner (Matcher B), who had previously played one of four roles: a silent side participant (seated next to the Director during her interactions with Matcher A), an omniscient bystander (watching and listening on a monitor in a separate room), an overhearer (seated behind the Director in such a way that they could hear the director and matcher A's conversation but could not see any of the referents), or a naïve participant (seated outside the experimental room engaged in a separate task, and so unable to see or hear any of the conversation).

On the first round following the changeover, Directors were fastest and used fewest words with former side participants, followed by omniscient bystanders; they were slowest and used most words with overhearers and naïve participants. They also produced significantly more indefinite references (and correspondingly fewer definite references) when Matcher B had been a simple bystander or naïve participant than a side participant or omniscient bystander. These results are consistent with Directors making different assumptions about the common ground that they shared with Matcher B on the basis of participant role. When Matcher B had been a side participant or omniscient bystander, Directors treated them similarly to Matcher A. In contrast, Directors treated overhearers in a similar manner to naïve participants, assuming little or no common ground. Thus, although overhearers had been able to hear descriptions, Directors acted as if this information was insufficient for successful reference without knowledge of the referent that each description was anchored to.

In sum, there is evidence that adult speakers are sensitive to variations in the information that they share with their addressees, and assume different levels of common ground depending on their addressee's participant role in previous discourse. Although there may be some leakages (e.g., failures to initially accommodate common ground during the earliest stages of processing; Horton and Keysar, 1996; Lane and Ferreira, 2008), adults tend to produce referential expressions that reflect these assumptions, with respect to the semantic content of their referring expressions (e.g., use of alternative conceptualizations), the amount of information they provide (e.g., shorter vs. longer referring expressions), and the form in which they express this information (e.g., use of definite vs. indefinite referring expressions).

Does children's referential communication similarly reflect their beliefs about what their partner is likely to understand? Certainly, children appear to be aware from an early age that people may have different knowledge from their own (e.g., Perner et al., 1987; Astington and Gopnik, 1991), and reflect this in their non-verbal communicative behavior (e.g., pointing and gesturing; Perner et al., 1987; Liszkowski et al., 2008). But is this awareness reflected in their language use, and what kinds of evidence are their beliefs about shared and unshared knowledge based on?

Some studies have shown that children, like adults, adapt their language production to reflect beliefs about their addressees' likely knowledge based on community membership. For example, children younger than five adapt the grammar and vocabulary of their utterances depending on their addressee's identity (e.g., producing less complex grammar and vocabulary when addressing a baby or a child than an adult; Shatz and Gelman, 1973; Sachs and Devin, 1976; Hansson et al., 2000; Hoff, 2010). This is consistent with a coarse degree of audience design that does not require detailed modeling of an addressee's knowledge, but can be based on broad distinctions (e.g., Galati and Brennan, 2010).

Children also show sensitivity to common ground based on past and present physical co-presence, though their ability to accommodate this information in their referential communication varies. Many studies have suggested that children are poor at producing unambiguous referential expressions to pick out one object from a complex array of objects with similar characteristics until well into school age (e.g., Glucksberg et al., 1966; Krauss and Glucksberg, 1969; Dickson, 1982; Deutsch and Pechmann, 1982; Lloyd et al., 1995, 1998). For example, Deutsch and Pechmann (1982) found that half of 6-year-olds (and a fifth of 9-year-olds) were unable to produce unambiguous referring expressions on their first attempt (e.g., saying the “*red one”* in a context involving several red objects), although they were responsive to their addressees' feedback.

Equally, Anderson et al. (1991) found that 7- to 8-year-olds (and 9- to 10-year-olds to a less marked degree) in route-giving dialogues that involved mismatching maps tended to inappropriately introduce new referents using definite references. Thus younger children presupposed that referents were shared with their addressees, rather than collaboratively establishing their shared status and a referential pact for how to refer to them (and their addressees were equally poor at providing feedback when referents were not in fact shared).

Such difficulties have been interpreted in terms of egocentric processing (Piaget, 1959). However, they may also reflect children's difficulties in determining relevant dimensions of contrast (e.g., Sonnenschein and Whitehurst, 1984). Recent studies have shown that by five, children can produce referring expressions whose content reflects the information that the child believes to be in perceptual common ground when the context makes it easier for the child to discriminate privileged from mutually shared knowledge. Hence 5-year-olds are more likely to produce an adjective to unambiguously pick out an object when there is a competitor object visible to both the child and their addressee than when the competitor is visible only to the child (e.g., Nadig and Sedivy, 2002; Bahtiyar and Küntay, 2009; Nilsen et al., 2009). Matthews et al. (2006) found that 3- and 4-year-olds also adapted the form of their referring expressions, such that their choice of (more informative) lexical NPs (e.g., “*The clown”*) vs. (less informative) pronouns (e.g., “*he”*) to refer to an entity was affected by whether the referent was visible to the addressee or not, although they still frequently failed to do so (e.g., 4-year-olds inappropriately produced pronouns on a third of trials where the referent was visually inaccessible).

In addition, older children adapt their referential behavior on the basis of previous physical co-presence, suggesting that in this age group the ability to engage in audience design based on shared physical context is not contingent on the context being concurrently available for consultation. Sonnenschein (1988) found that 6- to 9-year-old children produced referential expressions that contained more (possibly redundant) information when pretending to describe a toy for a stranger or friend with no shared experience than for a friend with whom they shared a common experience. Taken together, these results suggest that by school age, children are able to assess common ground based on past and current physical co-presence to at least some extent. Moreover, these assessments may affect both the amount of information provided in, and the form of, children's referential expressions, although they may not do so consistently and children's referential expressions may not always be optimal (e.g., in terms of redundancy).

There has been much less research on children's assumptions about common ground based on linguistic co-presence, and the extent to which these constrain referential processing. Unlike common ground based on concurrent physical co-presence, where the relevant context is available for consultation, common ground based on linguistic co-presence requires the child to be able to maintain and continuously update relevant information in memory. As such, it might be both more complex and more effortful to track. In comprehension, Matthews et al. (2010) found that 3- and 5-year-olds were slower to pick up and move an object when their partner referred to it using a different name (e.g., “*truck”*) than she had previously used to refer to it (e.g., “*car”*), than when a different partner, who had not previously named the object, referred to it using the new name (cf. Metzing and Brennan, 2003; Shintel and Keysar, 2007; Brown-Schmidt, 2009a). Graham et al. (2014) replicated these effects when the referential pact violation related to use of an adjective (e.g., “fluffy dog” vs. “spotted dog,” for a dog that was both fluffy and spotted), rather than different conceptualizations of the object at a categorical level. These results suggest that in comprehension, even pre-school children are sensitive to linguistic common ground, and specifically the referential pacts that they and a particular partner have established in previous discourse.

However, although these results suggest that children track the linguistic common ground that they have established with a partner, and are able to use this information to constrain comprehension by the age of four, children do not appear to use linguistic common ground to guide their production of referring expressions until later in development. Köymen et al. (2014) had 4- and 6-year-old children describe objects to a partner, and then describe the same objects in a different visual context to the same or a different partner. Six-year-olds were sensitive to whether or not they had previously established relevant referential pacts with a partner: If they had, they re-used the referring expression they had previously (tacitly) agreed; if they had not, they chose the referring expression that was most appropriate given the context of the array. Thus, their referential choices reflected audience design based on linguistic common ground. In contrast, 4-year-olds consistently produced referring expressions that were appropriate given the visual context, and showed no sensitivity to whether or not they and their addressee had previously established relevant linguistic common ground.

Other evidence suggests that children's ability to accumulate and use linguistic common ground appropriately continues to develop over a prolonged timecourse. Studies involving tasks in which children must communicate interactively about complex domains (e.g., maps with mismatching landmarks, mazes that involve complex spatial arrays) show that school-aged children experience difficulties in accurately modeling their partner's knowledge and responding to feedback up to the age of 11 and beyond (Anderson et al., 1991, 1994; Garrod and Clark, 1993). Garrod and Clark (1993) found that pairs of 7- to 8-year-olds sometimes converged on the same referring expressions, but without the same reference (e.g., both using *where you/I started*, but to refer to different locations), suggesting that their choice of referential expression was not based on a representation of common ground that included the crucial connection between a referring expression and its referent. Moreover, Anderson et al. (1994) found that even at the age of 13, a substantial minority of children performed no better than 7- to 8-year-olds. Clearly, children's ability to accumulate and flexibly exploit common ground when they speak in dialogue does not reach full maturity for many years.

Overall, the evidence suggests that, like adults, children maintain a representation of the language that they have previously used with a particular conversational partner, and that this model of linguistic common ground affects their referential processing to at least some extent from a young age, although the ability to use this information appropriately during the production of referential expressions appears to continue to develop into the teen years. But at what age do children develop a mature understanding of the accumulation of linguistic common ground? In particular, when do they become sensitive to participant roles, and understand that people accumulate common ground differently based on their participant role within a dialogue? All previous research has focused on how children use common ground accumulated within a dyadic dialogue involving just a speaker and an addressee. Although this research casts light on children's assumptions about common ground between speakers and addressees, it is not informative about children's awareness of the more general relationship between participant roles and the establishment of shared knowledge, in other words that listeners might develop shared knowledge with the speaker differently depending on whether they are licensed participants in the conversation or not.

The data from dyadic dialogues is compatible with children having an adult-like understanding that when a speaker proposes something and the addressee accepts it, the speaker's proposal becomes part of the linguistic common ground of all participants. But it also compatible with children having an impoverished understanding of the accumulation of linguistic common ground based on a simple distinction between having been the addressee of a particular utterance or not, or alternatively on having been present when something was said or not. In the former case, children might wrongly assume that someone who had previously been a side-participant would not have access to the language that was used in that conversation (or its interpretation); in the latter case, children might understand that an addressee who was not previously present would not have access to the language that was used in that conversation (and its interpretation), but they might wrongly assume that an overhearer who had been present during that conversation would also have access to it.

To distinguish these alternatives, we carried out an experiment in which eight- to ten-year old children played a tangram-description and -matching task with a partner, as in Wilkes-Gibbs and Clark (1992). One child was designated the Director, and played the game with another child (Matcher A) over four rounds (A1–4); the same Director then played the same game, using the same tangrams, with a second child (Matcher B; rounds B1-4). We manipulated Matcher B's participant role during the first four rounds, in order to vary the linguistic common ground shared by the Director and Matcher B during their subsequent interaction.

In the *side-participant* condition, Matcher B was seated next to the Director (and had the same view of the Director's tangrams as the Director) throughout the Director's rounds with Matcher A. Thus, Matcher B was able to hear all the references made and also verify whether these references were successfully resolved (through Matcher A's responses and the final outcome of each round). In the *overhearer* condition, Matcher B was seated in the same room but approximately 2 meters behind the Director with her back to the Director and Matcher; she could therefore hear references and exchanges with Matcher A, but could not see either player's tangrams, hence which tangram was being referred to. In the *naïve participant* condition, Matcher B was seated outside the experimental room; the Director and Matcher B therefore shared no common ground. Following Wilkes-Gibbs and Clark (1992), our primary measures were the total time taken for Directors and Matchers to match the set of tangrams each round, the number of correctly placed tangrams (measures of collaborative communicative success), and—in order to assess Directors' initial audience design based on their *a priori* beliefs about the Matcher's knowledge—the mean number of words per tangram that Directors used in their initial utterances before they received any formative feedback from the Matcher. As additional measures, we examined Directors' use of definite or indefinite reference (an index of whether Directors believed reference to be shared) and number of hedges (an index of their commitment to a particular conceptualization for a referent) in their initial utterances, as well as the number of basic exchanges (where the Director described a tangram and the Matcher immediately accepted this description; an index of the adequacy of the Director's audience design from the Matcher's perspective, i.e., whether the Matcher found the Director's initial description sufficient to identify the tangram).

Given previous findings that school-aged children are sensitive to the accumulation of linguistic common ground with an addressee, we expected that rounds A1–4 would show the same pattern as found in adults (Krauss and Weinheimer, 1964; Clark and Wilkes-Gibbs, 1986), specifically a tendency toward greater efficiency and shorter, definite descriptions that the addressee immediately accepts, which is assumed to reflect the exploitation of common ground accumulated with the partner over the course of the interaction.

However, our main interest is Directors' behavior in round B1, when interacting with a new partner. If children have an adult-like expectation that all participants within a dialogue (whether silent or actively involved) assume responsibility for their part in the collaborative process, then we would expect the same pattern as Wilkes-Gibbs and Clark (1992) found in adults. When playing with a former side-participant, the Director should assume that Matcher B has accumulated as much common ground during rounds A1–4 as both the Director and Matcher A. She should therefore assume that Matcher B has access to the referential pacts that she established with Matcher A, and so tend to use shorter, definite references with few hedges, and her descriptions should tend to be immediately comprehensible to Matcher B (yielding the same pattern of basic exchanges as with Matcher A). We would therefore expect the Director and Matcher B to take a similar amount of time and to have a similar level of accuracy as the Director and Matcher A did on round A4.

When playing the game with a former overhearer, the Director should assume that although she may have heard the linguistic expressions that were used, she would not have grounded their reference, and therefore does not have access to the referential pacts that she had established with Matcher A. She should therefore treat overhearers in the same way as naïve participants (as in Wilkes-Gibbs and Clark, 1992), yielding longer and more informative descriptions than on round A4, with more indefinite references and more hedges. Because she has not yet established a referential pact with Matcher B, we might expect that Matcher B would be less likely to find her initial description comprehensible, resulting in slower times, fewer basic exchanges, and lower accuracy than in round A4.

If however children have a non-adult-like understanding of the way in which linguistic common ground is accumulated, then we would expect a different pattern. If they make a simple distinction based on having been the addressee of a particular utterance or not, then in all three conditions they should treat their addressees as if they had no access to linguistic common ground, using longer and more informative descriptions, with fewer definite descriptions and more hedges, than round A4.

If instead children make a simple distinction based on having been present when something was said or not, then they should treat side-participants and overhearers (both of whom were present during rounds A1–4) differently from naïve participants (who were not). In that case, Directors should produce similar descriptions in the side-participant and overhearer conditions as on round A4, but in the naïve participant condition they should produce longer and more informative indefinite descriptions with more hedges. Because Directors would be erroneously overestimating addressees' knowledge in the overhearer condition, we might expect that accuracy in this condition would be reduced compared to A4 (and total time might be increased).

These predictions are based on the assumption that children's beliefs about linguistic common ground will be manifested in the same ways as in adults. However, the literature reviewed above shows that children may sometimes show audience design with respect to some aspects of language (e.g., use of lexical NPs vs. pronouns) but not others (use of definite vs. indefinite NPs). It may therefore be the case that children will show effects on some measures but not on others. Such a pattern would be informative about the extent to which children and adults manifest common ground in their linguistic behavior in the same way.

## Methods

### Participants

Seventy-two children aged between 8 and 10 years (mean: 9 years 7 months) recruited from a junior school in Nottinghamshire, UK, participated in the experiment (i.e., 8 groups per condition). This study was approved by the University of Edinburgh Psychology Department Ethics committee. Parents provided informed written consent for children's participation, and children provided verbal consent.

### Materials

The experimental items were eight tangrams taken from Wilkes-Gibbs and Clark (1992). Each tangram was printed in black on cream card (15 by 20 cm) and was laminated. Nine copies were made of the remaining eight tangrams to form the experimental sets; one copy was used by the matcher, and eight copies were used by the director (one set for each of the four rounds with each matcher). The tangrams in each of the director's sets were placed in numbered envelopes in a randomized order. Two further tangrams were used for demonstration and practice purposes.

To engage children with the task, we also provided a cardboard pyramid with a “jungle adventurer” theme; if children correctly matched four or more tangrams in a round, they could move an adventurer figure up a level on the pyramid. To ensure that children acting as Matcher B in the overhearer condition remained focused, and to give them a defined role (so that they were not perceived as an eavesdropper), we also prepared a handout featuring four pyramids, each with eight levels, for Matcher B to color in when they thought the Director and Matcher A had matched one tangram. We also prepared a booklet containing three mazes and games with a “jungle adventurer” theme, to occupy children who were not currently engaged in the game (Matcher B in the naïve participant condition for rounds A1–4; Matcher A in all three conditions for rounds B1-B4).

### Design

The experiment used a 3 × 2 mixed design, with Participant Role (side participant, overhearer or naïve participant) as a between-subjects factor and Round (A1 and A4, or A4 and B1) as a within-subjects factor.

### Procedure

Groups of three children were taken into the experimental room and told that they would play a “jungle adventurer” game, in which they would match ancient symbols to crack a secret code and reveal hidden treasure. Groups were randomly allocated to one of the experimental conditions. The children drew lots to decide roles. The Director and Matcher A took their seats, and Matcher B sat next to the experimenter where she could observe the table. A table divider in the middle of the table prevented Director and Matcher from seeing each other's cards. The children were told that they would play the game in two stages. First, the Director would describe the symbols in each envelope to Matcher A, so that the Matcher could put them in the same order; they could talk as much as needed to match the figures quickly and accurately. The Director and Matcher A would do this for four envelopes, all of which included the same symbols but in a different order. The Director would then do the same with Matcher B for a further four rounds.

One tangram was used as an example to familiarize the children with the figures; a second was used as a practice, to ensure that the Director provided sufficiently detailed descriptions. After the practice, Matcher B was informed of his role (in earshot of the director) before being taken to his corresponding position as a side participant, overhearer or naïve participant. Side participants were told that they would be able to see and hear what the Director was doing in the game, although they would not be playing it yet themselves. Overhearers were told that they would not be able to see anything but that they would be able to hear; they were also given the task of monitoring the Director and Matcher A's progress by coloring in levels on the pyramid sheet. Naïve participants were told that they would not be able to hear or see anything as they would be completing the activity booklet outside the room.

The Director opened the first envelope and laid out the cards in order. The Director and Matcher A then began their four rounds. After each round, the experimenter checked the accuracy of the card positions, and provided feedback about how many were correctly placed. After the Director and Matcher A had completed their four rounds (A1–4), Matcher B took the place of Matcher A (and in all conditions Matcher A took the overhearer's seat and was given the activity booklet to complete).

The children's interactions were audio-recorded using a tape recorder. The experiment took approximately 45 min to complete.

### Scoring

All rounds with Matcher A and Matcher B were timed from start to finish, using a stopwatch. Success was measured at the end of each round, by counting how many tangrams the children had correctly matched and converting this to a percentage.

Rounds A1, A4, and B1 were transcribed by the second author, and were independently coded by two coders who were ignorant of the experimental hypotheses (Cohen's kappa, a measure of inter-coder reliability, is reported below; in all cases, there was very high agreement). Disagreements were resolved by discussion. The dependent variables were based on the director's initial descriptions, before they received any feedback from the matcher. Feedback was classified as any sort of interruption or interaction (e.g., a question or contribution) that led to modification by the director; backchannel responses (e.g., *yeah*) that encouraged to the director to continue were not classified as feedback. Given that the initial description could only have been influenced by the director's *a priori* beliefs about their matcher's level of knowledge, this was judged to provide a more accurate and uncontaminated measure of audience design based on assumptions about linguistic common ground.

We recorded the *mean number of words* that the director used to introduce each figure before feedback from the matcher was recorded. Following Wilkes-Gibbs and Clark (1992), we coded directors' initial references as *definite reference* if they included the form *the x, this/that x*, or *the one with x*, or no article at all (e.g., *the next one is x*), and as *indefinite reference* if they included the form *a/an x.* (Other references were descriptive, e.g., *it has an X*; Cohen's kappa = 1). We further measured the number of *hedges* that directors produced, focusing on four specific forms: “*sort of,” “kind of,” “a bit,”* and “*-ish”* (Cohen's kappa = 0.985). We note that children also produced very high numbers of another type of hedge, namely *like* (e.g., *the next one's got erm two like half triangles*). Although these hedges are potentially highly informative, many examples could not be reliably discriminated as hedges vs. expressions of similarity (e.g., *it's got like a leg*), and we therefore did not code their use. Finally, we recorded the number of *basic exchanges* between directors and matchers. An exchange was coded as a basic exchange if the matcher immediately accepted the director's initial description without refashioning it in any way, so that the director immediately continued to the next tangram (Clark and Wilkes-Gibbs, 1986; Cohen's kappa = 0.991). We give examples of each coding category below.

1a. Definite reference: *The seal*
1b. Indefinite reference: *A man sat with no arms and no legs, like he's sat down.*
1c. Hedge [underlined]: *Like a head* *kind of* *triangle thing*
1d. Basic exchange: Director: *Zombie*
              Matcher: *Yeah*

## Results

We analyzed seven dependent variables: Mean total time (in seconds) per round; mean number of tangrams successfully identified (out of eight) per round; mean number of words per tangram in the Director's initial description per round; frequency of a definite referring expression in the Director's initial description per round; frequency of an indefinite referring expression in the Director's initial description per round; frequency of a hedge expression in the Director's initial description per round; and frequency of a basic exchange per round. Twenty-five data points (i.e., references to tangrams) were excluded because the Director did not refer to the relevant tangram (all involved the final tangram in a round, where the correct tangram could be identified by elimination).

We used mixed effects models to analyze the data. When the dependent variable was continuous, we modeled the response using linear mixed effects models, and when the dependent variable was binomial (basic exchange vs. not a basic exchange), we modeled the responses using logit mixed effects models (Baayen et al., 2008; Jaeger, 2008). For each binomial model, we were interested in predicting the probability of a positive response in the different conditions (i.e., that the children used a basic exchange). For all analyses there were two fixed effects (Participant Role and Round). Participant Role had three levels (naïve participant vs. side participant vs. overhearer), and Helmert coding was used to explore how the presence of Matcher B affected the Director's referring behavior. The first contrast compared the naïve participant condition, where Matcher B was not present during rounds A1–A4, to the mean of the overhearer and side participant conditions, where Matcher B was present during rounds A1–A4. A second contrast compared the overhearer and side participant conditions. Round had two levels for each analysis (A1 vs. A4 and A4 vs. B1); deviation coding was used to contrast each level. Full random effects models would not converge, so Round was removed from the random effects structure. Only significant (or marginal; *p* < 0.1) results are reported.

To confirm whether children showed the same patterns as found in previous research on adults when repeatedly describing the same referents to the same partner (e.g., Krauss and Weinheimer, 1964; Clark and Wilkes-Gibbs, 1986; Wilkes-Gibbs and Clark, 1992), we began by comparing rounds A1 and A4. Note that in these analyses, differences between Participant Role conditions would reflect incidental differences (e.g., in individual Directors' communicative skills), because the Participant Role manipulation was irrelevant at this stage. Any such differences in rounds A1–3 would moreover be irrelevant to our key questions, which hinge on differences between the final round with Matcher A (i.e., A4) and the first round with Matcher B.

However, our primary interest was in Directors' different assumptions about linguistic common ground with Matcher B as a function of Matcher B's previous participant role during rounds A1–4 with Matcher A. Hence the main comparisons of interest are those examining Directors' changes in behavior between their final round with Matcher A (A4) and their first round with Matcher B (B1).

### Total time taken per round (Table 1)

#### Rounds A1–A4

The model comparing Rounds A1 and A4 revealed a significant main effect of Round (β = −178.13, *SE* = 23.6, *t* = −7.54, p_*z*_ < 0.001^1^): Round A4 was completed faster than A1 (For this and all other linear mixed model analyses, *p*-values were calculated using a normal approximation). There was a marginal interaction between Participant Role and Round when naïve participant was contrasted with the two other conditions (β = 92.25, *SE* = 50.1, *t* = 1.84, p_*z*_ = 0.07): There was a greater reduction between A1 and A4 in the naïve participant condition (240 s) than the mean of the other two conditions (147 s). However, a model that included simple main effects for only round A4 showed that there was no difference between Participant Role conditions in round A4 (both p_*z*_ > 0.30).

**Table 1:** **Mean total time taken (sec) and percentage of tangrams correctly matched, by Round and Participant Role; standard deviation is in square brackets**.

	**Naïve Participant**			**Overhearer**			**Side-Participant**		
	**A1**	**A4**	**B1**	**A1**	**A4**	**B1**	**A1**	**A4**	**B1**
Total time (sec)	349 [91.7]	109 [57.7]	315 [190.4]	293 [166.5]	118 [48.4]	219 [165.8]	256 [115.7]	137 [78.6]	115 [87.0]
% correct	70.3 [30.6]	84.4 [18.6]	75.0 [22.2]	56.3 [32.7]	64.1 [27.1]	53.1 [25.7]	42.2 [28.3]	70.3 [29.1]	82.8 [20.0]

A second set of analyses examined whether there was a reduction in time across rounds A1–A4 in each Participant Role condition. For these analyses, rounds A1, A2, A3, and A4 were included in a model and Round was coded using polynomial coding. There was a significant linear trend for each Participant Role, with A1 being the slowest round and A4 being the fastest (all p_*z*_ < 0.01).

#### Rounds A4-B1

The model comparing rounds A4 and B1 with Helmert contrasts between the naïve participant condition and the other two conditions, and between the overhearer vs. side participant conditions, revealed a significant main effect of Round (β = 95.17, *SE* = 27.0, *t* = 3.53, p_*z*_ < 0.001); overall, round B1 was completed more slowly than A4. There was also a significant interaction between Participant Role and Round when naïve participant was contrasted with the other two conditions (β = −165.7, *SE* = 57.3, *t* = −2.89, p_*z*_ < 0.01), and a marginal interaction for overhearer vs. side participant (β = −122.63, *SE* = 66.1, *t* = 1.85, p_*z*_ = 0.06). Round B1 was 206 and 101 s slower than A4 when the matcher was a former naïve participant or overhearer respectively, but 22 s faster than A4 when the matcher was a former side participant.

A model that included simple main effects for round B1 showed a significant difference between the naive participant and the other two conditions (β = −145.3, *SE* = 53.2, *t* = −2.73, p_*z*_ < 0.01); and a marginal difference between overhearer and side participant (β = 101.73, *SE* = 54.8, *t* = 1.86, p_*z*_ = 0.06). Round B1 was slower when Directors were describing to a naïve participant than when they were describing to an overhearer or side participant. Directors were also slower when Matcher B had been an overhearer than when they had been a side participant.

### Number of tangrams correctly matched (Table 1)

#### Rounds A1–A4

The model comparing accuracy on rounds A1 and A4 revealed a significant main effect of Participant Role for naïve participant vs. the other two conditions (β = −1.50, *SE* = 0.66, *t* = −2.27, p_*z*_ < 0.05); there were more correctly matched tangrams in the naïve participant condition than in the other two conditions. There were no differences between the overhearer and side participant conditions. There was also a main effect of Round (β = 1.33, *SE* = 0.54, *t* = −2.49, p_*z*_ < 0.05; matchers correctly matched more tangrams in round A4 than A1. A model that included simple main effects for round A4 showed a marginal difference between the naïve participant and other two conditions (β = −1.41, *SE* = 0.76, *t* = −1.87, p_*z*_ = 0.06), with more correct tangrams in the naïve participant condition.

#### Rounds A4-B1

The model comparing Rounds A4 and B1 including Helmert contrasts showed a marginal main effect of Participant Role for naïve participant vs. the other two conditions (β = −1.01, *SE* = 0.57, *t* = −1.78, p_*z*_ = 0.07); participants correctly matched more tangrams in the naïve participant condition than the overhearer and side participant conditions (irrespective of round). A model analysing simple main effects for round B1 only showed a significant difference for the overhearer vs. side participant conditions (β = −2.19, *SE* = 1.03, *t* = −2.13, p_*z*_ < 0.05), with more correct tangrams in the side participant condition.

### Mean number of words per tangram (Table 2)

#### Rounds A1–A4

There was a significant main effect of Round (β = −11.83, *SE* = 1.29, *t* = −9.13, p_*z*_ < 0.001); Directors produced fewer words in their initial descriptions (prior to feedback) in A4 than in A1. There was a significant two-way interaction between Participant Role and Round when naïve participant was contrasted with the two other conditions (β = 8.39, *SE* = 2.75, *t* = 3.05, p_*z*_ < 0.01); Directors' initial descriptions reduced more from A1 to A4 in the naïve participant condition. There was also a significant two-way interaction between Participant Role and Round when overhearer was contrasted with side participant (β = −6.52, *SE* = 3.17, *t* = −2.05, p_*z*_ < 0.05); Directors' initial descriptions reduced more from A1 to A4 in the overhearer condition.

**Table 2:** **Mean number of words per tangram in Director's initial description, by Round and Participant Role; standard deviation is in square brackets**.

	**Naïve Participant**			**Overhearer**			**Side-Participant**		
	**A1**	**A4**	**B1**	**A1**	**A4**	**B1**	**A1**	**A4**	**B1**
Words/tangram	32.40 [19.3]	15.13 [12.4]	33.05 [23.4]	26.94 [17.6]	14.17 [7.4]	18.67 [10.1]	23.48 [16.6]	17.63 [14.2]	15.91 [11.7]

However a model that included simple main effects for round A4 showed no differences between the naïve participant and other two conditions, nor between overhearer and side participant (all p_*z*_ > 0.48); by A4, Directors in all conditions were producing a similar number of words to describe the tangrams in their initial descriptions.

#### Rounds A4-B1

The model comparing rounds A4 and B1 revealed a significant main effect of Round (β = −6.70, *SE* = 1.23, *t* = −5.45, p_*z*_ < 0.001); overall, Directors produced more words in their initial descriptions on their first round with matcher B than their last round with matcher A. There were also significant interactions between Participant Role and Round in the naïve participant condition contrasted with the other two conditions (β = 15.89, *SE* = 2.61, *t* = 6.08, p_*z*_ < 0.001), and for overhearer vs. side participant (β = −6.07, *SE* = 3.01, *t* = −2.02, p_*z*_ < 0.05). Directors used more words in their initial descriptions when addressing a new partner (Matcher B) who had been a naïve participant, and to a lesser extent when addressing a former overhearer. In contrast, Directors in the side participant condition used fewer words in their initial descriptions when describing tangrams to Matcher B for the first time than when describing the same tangrams to Matcher A for the fourth time.

A model analysing simple main effects for round B1 showed a significant difference only between the naïve participant condition contrasted with the other two conditions (β = −14.49, *SE* = 4.30, *t* = −3.37, p_*z*_ < 0.001). Directors produced more words when they knew that Matcher B was a naïve participant.

### Definite and indefinite references (Table 3)

#### Rounds A1–A4

##### Definite references

As no directors produced definite references in round A1, these data were not suitable for logit mixed effect models. All three Participant Roles showed an increase in definite references between A1 and A4. A model analysing simple main effects for round A4 showed no difference between participant role conditions (p_*z*_ > 0.96).

**Table 3:** **Frequency of definite and indefinite references in Director's initial description, and basic exchanges, by Round and Participant Role; percentage of total tangrams per round is in parentheses, and standard deviation is in square brackets**.

	**Naïve Participant**			**Overhearer**			**Side-Participant**		
	**A1**	**A4**	**B1**	**A1**	**A4**	**B1**	**A1**	**A4**	**B1**
Def. refs	0 (0.0%) [0.0]	20 (31.3%) [3.2]	3 (4.7%) [1.1]	0 (0.0%) [0.0]	15 (23.4%) [2.8]	5 (7.8%) [2.7]	0 (0.0%) [0.0]	24 (37.5%) [4.1]	22 (34.4%) [3.8]
Indef. refs	23 (35.9%) [2.5]	24 (37.5%) [3.3]	30 (46.9%) [3.1]	33 (51.6%) [2.7]	36 (56.3%) [3.4]	40 (62.5%) [3.0]	42 (65.6%) [3.4]	19 (29.7%) [3.0]	25 (39.1%) [3.0]
Basic exch.	32 (50.0%) [1.6]	50 (78.1%) [1.9]	41 (64.1%) [2.6]	44 (68.8%) [2.4]	52 (81.3%) [2.0]	43 (67.2%) [2.7]	42 (65.6%) [2.9]	46 (71.9%) [2.7]	53 (82.8%) [1.6]

##### Indefinite references

There was a significant main effect of Round (β = −0.70, *SE* = 0.29, *t* = −2.39, p_*z*_ < 0.05); children produced fewer indefinite references on A4 than A1. There were also two-way interactions between Participant Role and Round when the naïve participant condition was contrasted with the other two conditions (β = −1.28, *SE* = 0.59, *t* = −2.19, p_*z*_ < 0.05), and for overhearer vs. side participant (β = 3.13, *SE* = 0.75, *t* = −4.18, p_*z*_ < 0.001); Directors with a side participant initially produced the highest number of indefinite references but then substantially reduced their indefinite references between A1 and A4. Directors in the naïve participant condition produced the lowest number of indefinite references in A1, and both they and Directors in the overhearer condition showed little change across rounds. A model analysing simple main effects on round A4 showed only a marginal difference between the overhearer and side participant conditions (p_*z*_ = 0.08), with more indefinite references in the overhearer than side participant condition (36 vs. 19).

#### Rounds A4-B1

##### Definite references

The model comparing rounds A4 and B1 revealed a significant main effect of Round (β = 4.23, *SE* = 1.51, *Z* = 2.81, p_*z*_ < 0.001); Directors produced fewer definite references on their first round with matcher B than their last round with matcher A. The interaction with Participant Role was not significant, despite the greater number of definite references produced in B1 by Directors in the side participant condition. Closer inspection revealed that all definite references in the side participant condition (across all rounds) were produced by the same three directors.

##### Indefinite references

The model comparing rounds A4 and B1 showed a significant main effect of Round (β = −0.70, *SE* = 0.30, *Z* = 2.31, p_*z*_ < 0.05); Directors produced more indefinite references in round B1 than in A4, irrespective of participant role.

#### Hedges (Table 4)

##### Rounds A1–A4

The model revealed a significant main effect of Round (β = −0.23, *SE* = 0.05, *t* = −4.91, p_*z*_ < 0.001), indicating that the children produced fewer hedges on the fourth round with matcher A. There was also a significant interaction between Round and Participant Role for overhearer vs. side participant (β = −031, *SE* = 0.11, *t* = −2.71, p_*z*_ < 0.01): Directors reduced their number of hedges between Rounds A1 and A4 to a greater extent in the overhearer condition (23 vs. 3).

**Table 4:** **Frequency of hedges in Director's initial description, by Round and Participant Role; standard deviation is in square brackets**.

	**Naïve Participant**			**Overhearer**			**Side-Participant**		
	**A1**	**A4**	**B1**	**A1**	**A4**	**B1**	**A1**	**A4**	**B1**
Hedges	19 [3.9]	1 [0.4]	8 [2.1]	30 [5.1]	7 [1.5]	15 [3.0]	6 [2.1]	3 [0.7]	8 [2.4]

##### Rounds A4-B1

The model comparing rounds A4 and B1 revealed a significant main effect of Round (β = −0.11, *SE* = 0.04, *t* = −2.94, p_*z*_ < 0.01); Directors produced more hedges on B1 than on A4, irrespective of participant role.

#### Basic exchanges (Table 3)

##### Rounds A1–A4

The model revealed a significant main effect of Round (β = 1.17, *SE* = 0.28, *Z* = 4.14, p_*z*_ < 0.001); although there was a high proportion of basic exchanges even in round A1 (at least half of all descriptions in every condition), this number increased from A1 to A4. A model analysing simple main effects on round A4 showed no difference between participant role conditions on round A4 (ps_*z*_ > 0.19).

##### Rounds A4-B1

The model comparing rounds A4 and B1 revealed a significant interaction between Participant Role and Round for the contrast between the overhearer and side participant conditions (β = 2.23, *SE* = 0.76, *Z* = −2.92, p_*z*_ < 0.01); basic exchanges decreased when the director changed partners in the overhearer condition, but increased in the side participant condition. A model analysing simple main effects for round B1 showed a marginal difference only between the naïve participant condition contrasted with the other two conditions (β = 1.15, *SE* = 0.63, *Z* = 1.84, p_*z*_ = 0.06). Directors produced more words when they knew that Matcher B was a naïve participant.

## General discussion

Our experiment set out to examine 8-10-year-old children's assumptions about the accrual of linguistic common ground, and how this would affect their language use in a referential communication task. Specifically, we were interested in whether they would display an adult-like appreciation of differences in how common ground accumulates based on distinctions in listeners' participant roles. Previous research has focused exclusively on speaker-addressee pairings, and has suggested that children make assumptions that addressees have access to shared linguistic information in a way that people who have been absent from the conversation do not. However, such evidence does not demonstrate that children have a mature understanding of how differences in participant roles and the responsibilities associated with being a licensed participant in a conversation affect the accrual of common ground. Children might instead use simpler distinctions when assessing common ground, either overestimating its accumulation (by assuming that all listeners have access to it, irrespective of whether they are licensed participants), or underestimating its accumulation (by assuming that only addressees have access to it). We tested these possibilities by having children play a game in which they described the same set of tangrams repeatedly to another child and then described them again to a third child who had seen and heard the initial conversation, had only heard the conversation, or had neither seen nor heard the conversation.

We first consider the results from rounds A1 to A4 (before any change in partner), and their implications for children's accumulation of common ground in speaker-addressee pairings. The fact that Directors produced progressively shorter descriptions for the tangrams as they repeatedly described them to the same Matcher is consistent with previous research on adults (e.g., Krauss and Weinheimer, 1964; Clark and Wilkes-Gibbs, 1986). This shortening occurred for Directors' initial descriptions, prior to receiving any formative feedback from the Matcher, and therefore suggests that Directors were exploiting their knowledge of the linguistic common ground that they had built up with the Matcher to design, *a priori*, referring expressions that the Matcher would be able to understand. Analyses of Matchers' tangram-matching accuracy and time taken to complete each round suggest that Directors effectively exploited common ground in this way: Despite the shortening in initial referring expressions across rounds (from 27.6 words per tangram in A1 to 15.65 words in A4), tangram-matching accuracy increased (from 56.3 to 72.9%), and the time taken to complete each round decreased (from 299 to 121 s). Additionally, Matchers were more likely to accept the shorter initial descriptions in round A4 immediately without requiring further information, than the longer initial descriptions in A1 (77.1 vs. 61.5% basic exchanges respectively).

In other words, with increasing interaction with their partners, Directors produced shorter descriptions that were nevertheless communicatively more effective and more efficient. Our results therefore show that when 8–10-year-olds encounter novel objects with no conventional label, they are able not only to initially generate appropriate referring expressions for them in collaboration with their addressee, but also to subsequently draw on this shared linguistic information to design more concise but comprehensible references. These findings therefore extend previous research showing that children make use of referential pacts when referring to objects with conventional labels (Köymen et al., 2014).

Children also showed sensitivity to linguistic common ground in other aspects of their language. Their use of definite references (presupposing shared knowledge) changed across rounds. In round A1, where the Director and Matcher had no linguistic common ground, Directors never used definite references; in A4, where they had accrued common ground over the preceding three rounds, they used definite references on 30.7% trials. Children also produced fewer references that included expressions of uncertainty (hedges such as *sort of*) as they developed common ground with their partner, dropping from 18 hedges per round in A1 to 4 hedges per round in A4. Overall, then, the results of rounds A1–A4, prior to the manipulation of prior participant role, demonstrate that when interacting with a single partner, children of this age are able to track and use linguistic common ground, for at least some aspects of their language, in ways that enhance communication.

Unexpectedly, there were some differences between conditions in rounds A1–A4 (e.g., more correctly matched tangrams in the Naïve Participant condition), even though at this point the role of Matcher A was equivalent across conditions. It seems most likely that such differences reflect coincidental variations in individual children's performance rather than any effect of the experimental manipulation. As is clear from our results, and consistent with previous findings (e.g., Anderson et al., 1994), there were substantial individual differences in children's performance (e.g., in A1, the mean number of words in Directors' initial descriptions ranged from 7 to 55, and Matchers' tangram accuracy ranged from 0 to 100%).

We now turn to our main question of interest, namely children's assumptions about the accumulation of common ground based on differences in participant role. Our critical analyses therefore concerned changes in Directors' behavior between rounds A4 (their last round with Matcher A) and B1 (their first round with Matcher B). We were interested in whether children would show an adultlike pattern of treating former side participants in B1 in the same way as they had treated Matcher A in A4 (i.e., assuming equal access to common ground), in contrast to former overhearers and naïve participants; or would show a non-adultlike pattern, either treating all new partners alike (assuming no access to common ground, and yielding uniform differences between behavior on A4 and B1, irrespective of participant role), or treating both side participants and overhearers on B1 in the same way as they had treated Matcher A on A4 (with only naïve participants being treated differently). Our results suggest that although children have some understanding that linguistic common ground accumulates differently according to distinctions in listeners' participant roles, their understanding is not yet fully adultlike. They also suggest (in conjunction with analyses of rounds A1–A4) that the ways in which children draw on linguistic common ground in their language use differs from adults.

The primary evidence that children are sensitive to differences in participant role comes from analyses of the length of Directors' initial descriptions. These suggest that Directors made a tripartite distinction between the information available to former side participants, naïve participants, and overhearers. When addressing former side participants for the first time, they produced initial referring expressions that were very similar (and in fact, slightly shorter) than those that they produced when addressing Matcher A for the fourth time (A4: 17.63 words; B: 15.91 words). This is consistent with Directors assuming that side participants in a dialogue had access to the same linguistic common ground as addressees, and so could benefit from the same kind of concise referring expression.

Their ability to produce appropriate referring expressions in B1 on the basis of linguistic common ground accrued over rounds A1–A4 is supported by the fact that total time taken to complete the round did not increase when they first interacted with a new partner (indeed, it decreased by 22 s from A4 to B1) and at the same time tangram matching accuracy did not decrease (rather, increased by 12.5%); additionally, the number of turns in which Matchers were able to accept the initial description immediately did not decrease (rather, increased by 9.9%). Hence, Directors behaved as though they had the same common ground with former side participants as with former addressees; moreover, their ensuing referring expressions were communicatively effective, showing successful audience design on the basis of these assumptions.

In contrast, when Directors addressed former naïve participants for the first time, they produced initial referring expressions that were considerably longer than those that they had produced when addressing Matcher A for the fourth time (A4: 15.13 vs. B1: 33.05 words; this difference was significantly larger than the side participant/overhearer conditions). This result suggests that when Directors designed their referring expressions in B1, they assumed—prior to receiving any feedback from the Matcher—that a new Matcher who had been outside the room during the initial rounds required more information than Matcher A had required in A4; in other words, they assumed that naïve participants did not have access to the same common ground as addressees. Accordingly, the total time taken to complete the round increased by 206 s from A4 to B1, though tangram matching accuracy did not differ.

Directors also appeared to make a further distinction concerning the information available to former overhearers. Their initial referring expressions for Matcher B in round B1 were slightly longer than those for Matcher A in round A4 (A4: 14.17; B1: 18.67). The significant difference in the mean number of words per tangram in A4 vs. B1 in the overhearer and side participant conditions implies that Directors did not assume that former overhearers and former side participants had access to the same common ground. However, nor did they appear to treat former overhearers as having the same (lack of) knowledge as naive participants. The fact that Directors only slightly increased the length of their initial referring expressions suggests that they overestimated former overhearers' knowledge, and that this impacted negatively on communication. The total time taken to complete the round increased by 101 s, but more critically tangram accuracy in B1 was lower than in the side-participant condition; additionally, Matchers were less able to immediately accept Directors' initial referring expressions—indicating perceived understanding—in the overhearer condition than in the side participant condition (note that the two conditions did not differ on either measure in A4). It appears that Directors did not fully grasp the limited extent to which prior exposure to referring expressions alone, without simultaneous exposure to the reference of those expressions, was likely to facilitate subsequent comprehension.

In sum, evidence from the length of Directors' initial referring expressions suggests that children made largely accurate assumptions about the extent to which former naïve participants and former side participants had access to linguistic common ground, and designed their referring expressions accordingly, but also provides some suggestion that they were less accurate in gauging former overhearers' shared knowledge, with an apparent tendency to overestimate it. This pattern differs from that found in adults, who tend to treat addressees who previously had access to the linguistic content, but not the reference, of a prior dialogue in the same way as addressees who had no previous access to a prior dialogue (Wilkes-Gibbs and Clark, 1992).

However, this sensitivity to participant role is not borne out in other aspects of our data. Overall, Directors did not behave differently to former side participants, overhearers and naïve participants with respect to their use of definite and indefinite referring expressions, or hedges. Based on previous research on adults, we might have expected that the use of definites (implying shared knowledge) would decrease from A4 to B1 in the naïve participant condition relative to the side participant and overhearer conditions, and that the use of indefinites would conversely increase (Wilkes-Gibbs and Clark, 1992). We might also have expected the use of hedges (indicating provisionality prior to agreement on a referential pact) to increase (Clark and Wilkes-Gibbs, 1986; Brennan and Clark, 1996).

Although children did show differences on all three measures when interacting for the first time with a new partner (a lower use of definites, and a higher use of indefinites and hedges, in B1 than A4), these differences were uniform across conditions when the groups were considered as a whole. (However, we note that there was considerable variation between individual children in the use of hedges and definite references, suggesting that these aspects of language use might be particularly subject to individual differences in development; see also (Anderson et al., 1994; Nilsen et al., 2009), for further evidence of individual differences in dialogue skills). When considered alongside the evidence discussed above that children are nevertheless sensitive to differences in the accrual of linguistic common ground according to participant role, this pattern suggests that children do not accommodate these differences in their language in the same way as adults. In this study, assumptions about common ground manifested consistently in the length of children's referring expressions, but not the form of those expressions.

The conclusion that children and adults linguistically manifest common ground differently is supported by evidence from rounds A1 to A4. Although Directors showed increased use of definite expressions between rounds A1 and A4, definites still formed less than a third of their references in A4, and their use of indefinite expressions did not decrease between A1 and A4, remaining around two fifths of all references (41.6 vs. 41.2%). In contrast, Wilkes-Gibbs and Clark (1992) found that by the sixth round, Directors used definite references on 86.5% of trials and indefinite references on 4.2%. Thus, in adults' dialogue, there was a very strong relationship between the accrual of common ground and the use of definite expressions, whereas in our children this tendency was considerably weaker.

These results are consistent with other evidence suggesting that children do not use definite and indefinite reference in the same way as adults (e.g., Maratsos, 1974; Warden, 1976; Anderson et al., 1991). Most such research has found that children tend to overuse definite expressions, for example when first mentioning a referent that is unknown to the addressee. In these studies, children tend to incorrectly assume that their addressee has access to the same set of referents as themselves. Our study suggests that in a different context, where children knew that they had access to the same set of referents as their addressee but these referents did not have conventional names (and so could be conceptualized in multiple ways), children tended to underuse definite expressions, relative to adults. That is, they did not tend to use definite references that depended on (non-conventional) referential pacts (e.g., referring to *the rabbit*), although the shortening of referential expressions with increased common ground suggests that they were aware of, and exploited, these pacts (e.g., referring to a tangram in terms of its similarity to a rabbit). These results suggest that even at the age of 8–10 years, children's use of definite and indefinite referring expressions may differ from that of adults.

Finally, we consider other aspects of our results that suggest further disparities between children's and adults' referential processing in dialogue, focusing on rounds A1–A4 (to exclude any influences associated with changes in partner and participant role). Children's performance was consistently and considerably poorer than adults. Children's error rates ranged from 42.7% (A1) to 27.1% (A4). In contrast, Clark and Wilkes-Gibbs (1986) found error rates of around 2% in their studies (with a larger item set, which should have increased the likelihood of misidentification).

The high error rate is not surprising in itself, but it is indicative of the children's limited ability to detect and/or resolve misunderstandings. For an error to occur, Directors and Matchers must have terminated the process of presenting and accepting a reference inappropriately: The Director must have failed to detect that the Matcher had selected the wrong tangram, and the Matcher must have failed to realize that the Director was referring to a tangram other than the one they had selected. That is, they both inaccurately believed that the Matcher had understood the Director correctly, and therefore allowed the dialogue to move on. Thus although Matchers' increasingly accurate and faster performance across rounds in response to progressively shorter initial descriptions demonstrates that the Director and Matcher were able to build up and exploit common ground to some extent, the relatively high error rate overall indicates that this ability was still immature and far from adultlike.

This conclusion is supported by evidence from the occurrence of basic exchanges in rounds A1–4. Clark and Wilkes-Gibbs (1986) found that with adult participants, basic exchanges occurred relatively infrequently in the first round of the task, where participants had to identify novel objects for which they had as yet established no common ground, but became highly frequent in later rounds once common ground had been established (round 1: 18%; round 4: 80%). Thus adult Directors and Matchers tended to be cautious in their assumptions of mutual understanding, and to initially exchange multiple turns to establish confidence that understanding had been successfully achieved. In contrast, our children showed very high levels of basic exchanges even in the very first round (A1: 61.5%). Clearly, in these trials Matchers believed that they had understood the Directors, and Directors believed that Matchers had understood them—the instructions, the structure of the game, and the feedback provided by the experimenter after each round all ensured that children were aware that the Matcher must identify and place in the appropriate position the specific tangram described by the Director—but the tangram accuracy data show that this belief was often incorrect.

These results are consistent with many previous findings suggesting that children may have difficulties both in evaluating their addressee's understanding and appropriately responding when acting as speaker, and in detecting their own failure to understand and/or appropriately requesting information when acting as addressee (e.g., Bearison and Levey, 1977; Ironsmith and Whitehurst, 1978; Whitehurst and Sonnenschein, 1981; Anderson et al., 1991, 1994; Garrod and Clark, 1993; Lloyd et al., 1998). Thus in the same way that children may tend to overestimate the information that they share with a partner, they may also tend to overestimate the occurrence of mutual understanding.

Our study focused on one age group, and as such we cannot draw conclusions about the way in which, or age at which, children might come to develop adult-like behavior. Previous research suggests that even at the age of 13, a substantial minority of children continue to show behavior that differs from that found in experiments involving adults (Anderson et al., 1994). (Note, however, that most such experiments involve a relatively narrow population of highly educated individuals, i.e., college students, whose performance may not be representative of the adult population as a whole). The development of relevant dialogue skills may in part be dependent on the maturation of aspects of cognition such as executive function, such as the ability to inhibit one's own perspective. Certainly, research on both child and adult dialogue has implicated inhibitory control and working memory in online perspective-taking (Epley et al., 2004; Brown-Schmidt, 2009b; Nilsen et al., 2009; Lin et al., 2010).

However, in our experiment, the fact that Directors produced longer descriptions with naïve participants in B1 shows that they were able to suppress their own knowledge appropriately, suggesting that executive function (specifically inhibitory control) may be less relevant to our results, though working memory may have played some role. It therefore seems likely that the development of adult-like behavior cannot be reduced simply to the maturation of executive functions, and instead involves the development of a more elaborated understanding of what information is and is not shared by speakers and addressees on the basis of previous discourse (e.g., whether speaker and addressee share the reference of a referring expression).

We suggest that the interactions that children experience may play an important role in shaping this developmental process. Many studies have suggested that experience of communication breakdown and its subsequent resolution through formative feedback from listeners—whether at first-hand or through observation—may play an important role in improving young children's performance in dialogue tasks (e.g., Robinson and Robinson, 1981, 1985; Deutsch and Pechmann, 1982; Matthews et al., 2007, 2012). In principle, all of the interactions that children experience could give them valuable evidence about the accrual of common ground under different circumstances. However, given that formative feedback depends crucially on the listener, and given that school-aged children– as our and other studies show—are not always adept at gauging their own understanding and providing informative feedback, it may be the case that interactions with more mature language users (adults and near-adults) play a particularly important role in developing relevant skills and understanding even into the early teen years.

In conclusion, this research investigated what inferences 8–10-year-old children were able to draw about their partners' knowledge on the basis of their participation in previous dialogue. Our results suggest that by this age, children have some understanding that the accumulation of linguistic common ground is affected by participant role. In particular, they assume that side participants in a dialogue build up linguistic common ground (and have access to this common ground in subsequent dialogues involving the same speaker), and that overhearers do not have access to this information to the same extent. These assumptions are reflected in the amount of information that they provide in their referring expressions. However, our results also suggest that children are not fully adult-like at this age in both their understanding and their linguistic use of common ground. Children appear to overestimate the extent to which listeners who overhear but do not participate in a dialogue accumulate common ground, and do not use definiteness to reflect linguistic common ground in the same way as adults. These results, together with evidence of other limitations in children's dialogue skills (e.g., overestimations of mutual understanding) provide further evidence that learning to use language successfully in interaction is a slow process that continues to develop until well into the school years.

## Funding

This research was supported by ESRC grant ES/F023359/1 awarded to JB and a British Academy/Leverhulme Trust Senior Research Fellowship awarded to HB.

### Conflict of interest statement

The authors declare that the research was conducted in the absence of any commercial or financial relationships that could be construed as a potential conflict of interest.

## Footnotes

^1^For this and all other linear mixed model analyses, p-values were calculated using a normal approximation.
